# Acute Lymphoblastic Leukemia following Lenalidomide Maintenance for Multiple Myeloma: Two Cases with Unexpected Presentation and Good Prognostic Features

**DOI:** 10.1155/2018/9052314

**Published:** 2018-02-12

**Authors:** Abdullah M. Khan, Jameel Muzaffar, Hermant Murthy, John R. Wingard, Jan S. Moreb

**Affiliations:** Division of Hematology and Oncology, Department of Medicine, University of Florida, Gainesville, FL, USA

## Abstract

Lenalidomide maintenance following autologous stem cell transplant (ASCT) is considered the standard of care for eligible patients with multiple myeloma (MM). A recent meta-analysis has provided additional evidence that lenalidomide maintenance is associated with a higher incidence of second primary malignancies, including both hematologic and solid malignancies. Acute lymphoblastic leukemia (ALL) as a second primary malignancy is rarely described in the literature. Herein, we describe two patients with MM treated with induction therapy, ASCT, and lenalidomide maintenance that experienced cytopenias while on maintenance. ALL was unexpectedly diagnosed on bone marrow biopsy. One patient was diagnosed on routine biopsy performed as part of requirements of the clinical trial. Both patients had B-cell ALL, without known poor risk cytogenetics, and were managed with standard induction therapies resulting in complete remission. We also reviewed the literature for similar cases of secondary ALL (sALL) in MM patients exposed to immunomodulatory drugs (IMiDs). In conclusion, persistent cytopenias in responding MM patients receiving IMiDs maintenance should be an indication for bone marrow biopsy. Patients develop sALL after median of 32.5 months (range, 20–84) from being on lenalidomide or thalidomide maintenance, often presenting with cytopenias, display low tolerance to chemotherapy, but remission can often be achieved.

## 1. Introduction

Over 30,000 new cases of multiple myeloma (MM) are expected to be diagnosed in 2017 [1]. Arguably, the standard of care for eligible patients includes autologous stem cell transplant (ASCT) [2–4]. However, since MM is rarely cured with ASCT, there has been considerable interest in maintenance therapy with a goal to prolong progression free survival (PFS) and overall survival (OS). Three major trials have evaluated the role of lenalidomide maintenance in this regard [5–8]. In February 2017, on the basis of the Cancer and Leukemia Group B (CALGB) 100104 and Intergroup Francophone of Myeloma (IFM) 2005-02 studies, the Food and Drug Administration expanded its indications for lenalidomide to include maintenance therapy for patients with MM following ASCT. Furthermore, a meta-analysis of the three trials demonstrated OS benefit of lenalidomide maintenance, supporting the argument that it should be considered a standard of care after ASCT [9].

Further detailed in the meta-analysis was the association between second primary malignancies (SPM) and lenalidomide maintenance. The hazard ratio for a second primary hematologic malignancy was 2.03 (*P*=0.015), and the hazard ratio for a second primary solid malignancy was 1.71 (*P*=0.032) [9]. In the updated analyses of the two studies mentioned above, total of hematological SPMs reported was 34 and among those 8 cases of B-cell acute lymphoblastic leukemias (ALLs) [10]. It is not yet clear how lenalidomide contributes to ALL pathogenesis. Herein, we describe two cases of B-cell ALL, who received lenalidomide on the CALGB 100104 study, with unique presentation, generally with good prognostic features and with good response to standard chemotherapy. We also review the literature for similar ALL cases associated with immunomodulatory drugs (IMiDs) maintenance.

## 2. Case Presentation

We report here our two cases of secondary ALL (sALL) after exposure to lenalidomide maintenance. In addition, we reviewed the literature for any previously published similar cases in PubMed. We extracted pertinent information in regards to patient age, gender, prior IMiD exposure, length of exposure to IMiD therapy, phenotype of sALL, cytogenetics abnormalities, treatment given for the sALL and outcomes. Descriptive statistics were used.

### 2.1. Case 1

A 53-year-old female presented with back pain and was found to have a sacral mass in May 2009. After a thorough evaluation, she was diagnosed with a solitary plasmacytoma that was treated with radiation therapy. She had an IgG lambda monoclonal spike that was monitored for over a year before she showed progression to active MM according to her MM markers and new symptomatic bone lesions. Treatment was started with bortezomib and dexamethasone. She was enrolled to CALGB 100104 (supported by ECOG and BMT CTN) and underwent single autologous stem cell transplantation (ASCT). Her disease status after ASCT was very good partial response and, per trial protocol, she was started on lenalidomide maintenance therapy. Due to low absolute neutrophil counts (<1000), she initially required frequent lenalidomide dose interruptions and adjustments per the clinical trial. Six years into her maintenance therapy, her MM was in complete remission but developed low white blood cell and platelet counts prompting holding lenalidomide. Due to incomplete count recovery, a bone marrow biopsy was obtained. Morphology and flow cytometry revealed 50% blasts which expressed CD19, CD33, CD34, CD79a, HLA-DR, PAX5, and TdT but did not express CD117 or MPO. Of note, <5% plasma cells were detected and showed polyclonal kappa and lambda expression. Cytogenetics revealed trisomies of chromosomes 8, 10, and 21, monosomy chromosome 20. Fluorescent in situ hybridization (FISH) showed MYC and IGH gene locus copy number gain. The patient was treated using the CALGB study 8811 protocol [11]. The patient had severe hepatotoxicity with peg-asparaginase prompting long delays in her therapy; however, she had complete remission according to repeat bone marrow biopsy done one month after starting induction chemotherapy. Her subsequent therapy was interrupted due to significant myelosuppression that lead to the shortening of her intensification therapy. Currently, she is 12 months from diagnosis, in complete remission from her ALL, on maintenance with 6-mercaptopurine (Purinethol), vincristine (Oncovin), methotrexate, and prednisone (POMP). Her myeloma has remained in stable complete remission as well.

### 2.2. Case 2

A 69-year-old female transferred oncologic care to the University of Florida in February of 2013. She was diagnosed with IgG kappa multiple myeloma in February 2011 with Durie–Salmon stage 3B due to lytic bone lesions and elevated creatinine. She was initially treated with bortezomib and dexamethasone before adding lenalidomide due to inadequate treatment response. She was enrolled onto the CALGB 100104 clinical trial and underwent tandem autologous transplantation (conditioning regimen with melphalan 200 mg/m^2^) followed by lenalidomide maintenance. At the time of evaluation in our institution, the patient had been on lenalidomide for 15 months. She had a hemoglobin content of 11.1 g/dL, platelets of 81,000/mm^3^, and WBC of 2000/mm^3^ with an absolute neutrophil count of 960. Chemistry revealed normal renal function and absence of hypercalcemia. Myeloma markers revealed hypogammaglobulinemia, an IgG kappa monoclonal spike of 0.5 g/dL, kappa 2.52 mg/dL, lambda 0.12 mg/dL, and a kappa/lambda ratio of 21. She had frequent lenalidomide dose adjustments or interruptions due to cytopenias. A required bone marrow biopsy, per the clinical trial, was obtained after cycle 23 of lenalidomide maintenance and revealed 1-2% of kappa restricted plasma cells. As part of the study requirements, a repeat bone marrow biopsy was obtained after three years of lenalidomide maintenance which revealed a hypocellular marrow with 25% abnormal B-lymphoblasts. Due to the unexpected results, a bone marrow biopsy was repeated with similar findings. The lymphoblasts expressed bright levels of CD34 and TdT, intermediate levels of CD19 and CD22, and diminished or absent expression of CD10, CD20, and CD45. Cytogenetics evaluation was normal but one cell had 46, XX, t(7;19). FISH testing was negative for BCR/ABL1, MLL, and RUNX1 gene loci fusion rearrangement as well as negative for chromosome 4 and chromosome 10 copy number changes. She was treated with hyper-CVAD [12] for 8 months followed by POMP maintenance. A postinduction bone marrow aspirate and biopsy showed a variably hypocellular marrow without overt morphologic evidence of B-cell ALL and ∼6% abnormal plasma cells. She has not required further treatment for her myeloma and remained in a stable very good partial response. She is 36 months from her ALL diagnosis in continuous remission.

## 3. Discussion

The 5-year relative survival rate has improved from 24% for patients diagnosed with MM between 1975 and 1977 to 51% in patients diagnosed between 2007 and 2013 [13]. This increased life expectancy has raised concerns about the risks associated with prolonged exposure to antimyeloma therapy, including lenalidomide maintenance following ASCT [14]. Among the risks is the development of hematologic and solid SPM. Initial observations suggested a predilection for hematologic SPM of myeloid origin including myelodysplastic syndrome, acute myeloid leukemia, and chronic myeloid leukemia. However, updated clinical trial data and a number of case reports reveal an increased risk of lymphoid malignancies as well [9, 14].

The two cases we presented here describe unique asymptomatic presentation, the ones having been diagnosed on routine bone marrow biopsy. Both patients had pancytopenia that was thought to be related to lenalidomide effects. Thus, secondary acute leukemia should be suspected in MM patients who develop unexplained persistent cytopenias. The other characteristics to note are that both of our patients did not display any of the known ALL bad prognostic features and were able to attain complete remission of their sALL with standard induction therapy. These two patients were most likely included in the recent updated analysis of the CALGB 100104 [8] which includes detailed description of all SPMs including the cases of B-cell ALL.

Eleven cases of ALL following IMiDs (lenalidomide and thalidomide) maintenance in the treatment of MM have been reported in the literature [15–21]. Only part of these reports made the connection to the IMiDs exposure.  Table 1 shows the summary of the findings from our 2 patients and 11 patients described in the literature. We can summarize that B-cell ALL is the dominant morphological type and the usual presentation is cytopenias and fatigue while on IMiDs. Six patients had thalidomide as the main IMiD. The median time of exposure to the IMiDs before developing sALL was 32.5 months (range, 20–84). There were no unique chromosomal abnormalities associated with this diagnosis. When performed, FISH studies for the known bad prognostic ALL gene abnormalities were usually negative. Tolerance for intensive ALL chemotherapy could be challenging in these cases, and 2 patients died of septicemia, while few patients gave up and most likely died without treatment. However, the best treatment of these patients is not yet clear. In general, secondary leukemias have worse prognosis and require allo-SCT for curative purposes. Only one of these patients had that treatment with good outcome so far. Occasionally, MM patients may have autologous stem cell products frozen from their planned treatment for MM which can be used for second ASCT as a consolidation in the treatment of their sALL. Indeed, one patient was treated that way successfully. Our two patients seem to have done well with standard chemotherapy including CNS prophylaxis but required significant modification of drug doses due to severe myelosuppression.

Cases of sALL have been described in MM patients that were not exposed to IMiDs and was attributed to the melphalan, other chemotherapy used [22, 23], or possibly disease-related immune deficiency. At least some of these cases were associated with MLL gene rearrangement [23] which seems to be associated more frequently with sALL that develop after non-IMiD type of standard chemotherapy, discussed below.

In general, most secondary acute leukemias described in the literature have been secondary acute myeloid leukemia (AML), while sALL is much less common. It is believed to represent just 1–3% of all ALL and up to 10% of all secondary acute leukemia [24–27]. There are no large studies comparing sALL with de novo ALL, and most of the information has been derived from small case series reported in literature. Patients with sALL tend to be in elderly patients and have a worse outcome. Most of the sALL are of B-cell type and tend to have some high-risk cytogenetics and molecular features such as 11q23 rearrangement (especially after exposure to topoisomerase II inhibitors), as well as BCR-ABL and complex chromosomal abnormalities [25]. Treatments for breast cancer and lymphoma, both Hodgkin's and non-Hodgkin's, are the most common cancers associated with development of sALL [26]; however, in one series a sizable proportion of sALL patients were not treated with prior chemoradiotherapy [24]. The median latency period to development of sALL varied between 12 and 27 months, depending on the type of first malignancy and prior anticancer therapy, with a range between 0.8 and 9 years [25]. The largest retrospective studies have shown that type of first malignancy, previous treatment, interval to diagnosis, and cytogenetic abnormalities do not appear to affect the overall survival in sALL patients [24–27]. Median overall survival has been shown to be around 8 months as compared to 11 months for de novo ALL, and after adjusting for other variables, sALL itself appears to be an independent risk factor with dismal survival at 1, 3, and 5 years (35%, 16%, and 6.8%, resp., versus 47%, 31%, and 21% in de novo ALL for the same time periods). Age has been the only other factor affecting survival, with worse outcomes seen with increasing age [26]. Recent study published by Rosenberg et al. [28] extracts data on sALL from the Surveillance, Epidemiology, and End Results (SEER) cancer registry program between 1988 and 2012 and reports 3% of 14,470 patients with ALL were ALL with antecedent malignancy. According to this study, the most common prior malignancies were breast cancer (21%), hematologic malignancies (18%), and male genital system (15%).

Our findings show that the median latency period for sALL post-IMiDs seems to be longer (32 months) than that reported after other chemotherapy, and no cases were reported before 20 months from exposure to one of the IMiDs. On the other hand, sALL after other chemotherapy can present as early as few months after chemotherapy [24–28]. Also, in many but not all cases of sALL after non-IMiDs chemotherapy, MLL gene aberrations are reported [29], while none of the IMiDs-associated sALL cases, with known cytogenetics/FISH tests (Table 1), had such abnormality. These findings suggest different pathogenesis and mechanism for development of sALL after IMiDs. The exact mechanism is not known, but hints can be sought from published data on lenalidomide and thalidomide mechanism of actions and effects, especially on B lymphocytes. All IMiDs were reported to reactivate the Epstein–Barr virus lytic cycle in resting memory B cells, contributing to increased immunosuppression and possible mechanism for the development of secondary Hodgkin's lymphoma in post-ASCT/lenalidomide maintenance setting [30]. Another potential effect of lenalidomide on B cells could be through its known effect on the cereblon protein, changing the substrate specificity of the CRL4 (cereblon) E3 ubiquitin ligase complex, such that the proteins IKAROS (encoded by *IKZF1*) and AIOLOS (encoded by *IKZF3*) are selectively ubiquitinated and degraded in multiple myeloma cells [31, 32]. IKAROS and AIOLOS transcription factors are critical regulators of B-cell function in mice and humans [33]. IKAROS plays key roles in human B-cell development and malignancy, which is further studied by another recent publication [34]. Lenalidomide was also reported to expand the absolute lymphocytic counts in peripheral blood of patients treated for myelodysplastic syndrome, with expansion of the regulatory T cells (Treg) [35]. Treg could potentially promote immune tolerance towards any abnormal clonal expansion. On the other hand, the effect of IMiDs on Treg in myeloma patients is complex and seems controversial [36]. Whether any of these effects of lenalidomide play a role in the development of B-cell sALL in MM patients is worth further investigation.

In conclusion, persistent pancytopenia on IMiDs maintenance in MM patients should be investigated with bone marrow biopsy since it could be caused by secondary B-cell ALL. Our report shows that sALL can be seen not only after lenalidomide exposure but also after thalidomide, thus it could potentially be a class effect. Caution should be used in using intensive induction chemotherapy in these patients due to intolerance and risk of fatal infectious complications. However, in general, complete remission can be achieved in most patients and it can be durable, but long-term follow up (>5 years) is needed in order to determine curability and the subsequent behavior and outcome of the MM as well.

## Figures and Tables

**Table 1:** Characteristics of patients with multiple myeloma who were exposed to IMiDs maintenance and developed acute lymphoblastic leukemia as a second primary malignancy.

Reference number	IMiD type	Time to Dx (mo)	Age (Y)/sex /presentation	Morphologic type	Cytogenetics/FISH	Treatment/outcome
[15]	Len^1^	20	62/F/pancytopenia	B cell (CD19, 20, 10, 22, 79a, TdT, IgMc+, and CD34−)	Normal	Death due to septicemia during induction
[16]	Len	2	66/M/fatigue, low WBC	B cell (CD45, 19, 22, 34+, CD79±, TdT+)	Trisomy 4	Death 8 mo with no response to induction with CHOP
	Thal	31				
[17]	Len^1^	30	59/M/fatigue, pancytopenia	B cell (CD10, 19+, CD79a, Pax5, and TdT±)	Deletion 20q	Linker regimen, alive > 1 y after allo-SCT in CR
[17]	Len	36	34/M/pancytopenia and circulating blasts	B cell (CD10, 19, 22, 34, 79a, 38+)	Chr 14 rearrangement	CALGB 8811, death 1 month after induction due to CVS and septicemia
[17]	Len^1^	84	53/M/leukopenia	B cell (CD19, 34, 79a, PAX5, TdT+)	Small population of tetraploid cells by FISH	Linker regimen, >1 y after 2nd ASCT in CR
[18]	Thal^1,2^	29	61/F/pancytopenia	B cell (CD19, 38+)	NA	CALGB 9111
[19]	Len^1^	4.5	65/F/dizziness	B cell (CD19, 22, 34, 79a+, HLA-DR+)	NA	No treatment, death after 14 mo
	Thal	32.5				
[19]	Thal	32	63/M/fatigue, cytopenias	B cell (CD10, 34+, HLA-DR, and CD33 dim)	NA	Lost to follow-up
[19]	Thal^1^	73	33/F/cough, fatigue and anemia	B cell (CD10, 34, 19, 22, 33, 79a+, HLA-DR+)	NA	No treatment, lost to follow-up
[20]	Thal	53	56/F/edema and dyspnea, pancytopenia	B cell (CD19, 20, 22, 10+, HLA-DR+)	Normal	Steroids only, DVT/PE, death within 10 days
[21]	Len	24–36	72/M/pancytopenia	B cell (CD10, 19, 20, 79a, and TdT+, CD34, 117, 38, 56−)	NA	Hyper-CVAD, POMP maintenance. CR for 26 mo, then relapse
Our case	Len^1^	23	69/F/none	B cell (CD34, 19, 22+)	t(7;19) in one metaphase	Hyper-CVAD, then POMP maintenance, in CR for 3 year
Our case	Len^1^	72	53/F/pancytopenia	B cell (CD19, 33, 34, 38, 79a+, PAX5+, TdT+)	+8, +10, +22, −20, and FISH showed MYC and IgH gene locus copy gain	Abbreviated CALGB 8811 due to intolerance, now on POMP maintenance, in CR for 1 year

^1^These patients had prior ASCT, while the others did not; ^2^developed ALL 3 years after stopping Thal maintenance.
