# Corrigendum to “Acute Lymphoblastic Leukemia following Lenalidomide Maintenance for Multiple Myeloma: Two Cases with Unexpected Presentation and Good Prognostic Features”

**DOI:** 10.1155/2019/1548610

**Published:** 2019-08-04

**Authors:** Abdullah M. Khan, Jameel Muzaffar, Hemant Murthy, John R. Wingard, Jan S. Moreb

**Affiliations:** Division of Hematology and Oncology, Department of Medicine, University of Florida, Gainesville, FL, USA

In the article titled “Acute Lymphoblastic Leukemia following Lenalidomide Maintenance for Multiple Myeloma: Two Cases with Unexpected Presentation and Good Prognostic Features” [1], the first name of the third author was given incorrectly as Hermant. The author's name should have been written as Hemant. The revised authors' list is shown above.
